# Host Immune Response to Mosquito-Transmitted Chikungunya Virus Differs from That Elicited by Needle Inoculated Virus

**DOI:** 10.1371/journal.pone.0012137

**Published:** 2010-08-12

**Authors:** Saravanan Thangamani, Stephen Higgs, Sarah Ziegler, Dana Vanlandingham, Robert Tesh, Stephen Wikel

**Affiliations:** 1 Department of Pathology, University of Texas Medical Branch, Galveston, Texas, United States of America; 2 Center for Biodefense and Emerging Infectious Diseases, University of Texas Medical Branch, Galveston, Texas, United States of America; 3 Sealy Center for Vaccine Development, University of Texas Medical Branch, Galveston, Texas, United States of America; Global Viral Forecasting Initiative, United States of America

## Abstract

**Background:**

Mosquito-borne diseases are a worldwide public health threat. Mosquitoes transmit viruses or parasites during feeding, along with salivary proteins that modulate host responses to facilitate both blood feeding and pathogen transmission. Understanding these earliest events in mosquito transmission of arboviruses by mosquitoes is essential for development and assessment of rational vaccine and treatment strategies. In this report, we compared host immune responses to chikungunya virus (CHIKV) transmission by (1) mosquito bite, or (2) by needle inoculation.

**Methods and Findings:**

Differential cytokine expression was measured using quantitative real-time RT-PCR, at sites of uninfected mosquito bites, CHIKV-infected mosquito bites, and needle-inoculated CHIKV. Both uninfected and CHIKV infected mosquitoes polarized host cytokine response to a T_H_2 profile. Compared to uninfected mosquito bites, expression of IL-4 induced by CHIKV-infected mosquitoes were 150 fold and 527.1 fold higher at 3 hours post feeding (hpf) and 6 hpf, respectively. A significant suppression of T_H_1 cytokines and TLR-3 was also observed. These significant differences may result from variation in the composition of uninfected and CHIKV-infected mosquito saliva. Needle injected CHIKV induced a robust interferon-γ, no detectable IL-4, and a significant up-regulation of TLR-3.

**Conclusions:**

This report describes the first analysis of cutaneous cytokines in mice bitten by CHIKV–infected mosquitoes. Our data demonstrate contrasting immune activation in the response to CHIKV infection by mosquito bite or needle inoculation. The significant role of mosquito saliva in these earliest events of CHIKV transmission and infection are highlighted.

## Introduction

Mosquitoes are a significant public health problem because of their ability to transmit a variety of arboviruses and also the causative agents of malaria and filariasis to susceptible humans (www.who.int/tdr/diseases). Mosquito-borne diseases continue to emerge and re-emerge [1], [2] as demonstrated by the recent chikungunya epidemics on Indian Ocean islands and in India since 2005. Chikungunya virus (CHIKV) is an *Alphavirus* belonging to family *Togaviridae*, which is transmitted predominantly by *Aedes aegypti* and *Ae. albopticus* (www.cdc.gov/ncidod/dvbid/Chikungunya). Both mosquito species occur over vast regions of the world, including the United States, southern Europe, and tropical regions of South America, Africa, and Asia posing the very real threat that new transmission cycles could be established in these regions [3]. Since 2005, CHIK fever has been identified in an unprecedented number of travelers returning home from epidemic areas to Europe, United States, Australia, and Japan [3], [4], [5], [6], [7]. Imported CHIKV infection in returned travelers paralleled the spread of the explosive outbreaks in the Indian Ocean islands and India. In 2006, CHIKV infections were detected in Singapore among travelers returning home after visiting India and Malaysia. Those sporadic imported cases preceded the 2008 chikungunya outbreaks in Singapore, demonstrating the potential for introducing this emerging viral infection into new areas and establishing a transmission cycle with competent local vector mosquito species [8]. Thus, there is a clear risk of importing CHIKV into new ecological niches through infected travelers returning from popular tourist destinations with CHIKV epidemics.

Human infections with CHIKV occur during blood feeding by infected *Aedes* mosquitoes. Mosquito saliva contains a repertoire of pharmacologically important proteins/factors that modulate host haemostasis, immune response and other defenses thus facilitating blood feeding and pathogen transmission [9], [10], [11], [12], [13], [14]. Previous studies have reported that mosquito bite enhances infection with Cache Valley virus (CVV) [15], West Nile virus (WNV) [16], vesicular stomatitis virus (VSV) [17], [18], and La Crosse virus (LACV) [19]. Exposure to the bites of uninfected *Ae. aegypti* exacerbated mosquito transmitted WNV infection [20]. Mosquito feeding skews host T-cell immune responses away from a T_H_1 to a T_H_2 phenotype [21], [22], [23], which subsequently creates an environment that favors arbovirus transmission and infection that would otherwise be neutralized by T_H_1 cytokines [21], [24]. We recently showed the dynamics of dermal T_H_1 and T_H_2 cytokine expression at the bite sites of *Ae. aegypti* and identified an *Ae. aegypti* salivary gland protein that causes T_H_2 polarization of host CD4+ T-cells [25].

The earliest events of CHIKV transmission by mosquitoes remain poorly understood. Current knowledge of CHIKV infection comes from studies of WNV, LACV, and CVV infections. Effects of mosquito saliva on dermal cell expression of cytokines after a CHIKV-infected mosquito bite are unknown; but characterization of these mosquito-host-pathogen interactions will result in a better understanding of the earliest events in the successful transmission and establishment of CHIKV infection. Here we describe the influence of CHIKV-infected mosquito bite on the cutaneous T_H_1 and T_H_2 cytokine responses and compare the responses to that following needle injection of CHIKV.

## Results

### Dermal cytokine responses to uninfected or CHIKV infected mosquito bites

**Figure 1:**
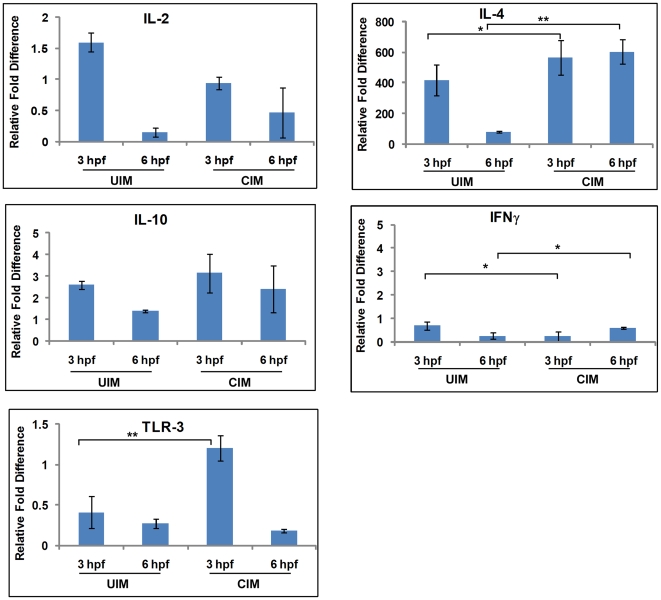
Comparison between uninfected mosquito (UIM) bites and CHIKV infected mosquito (CIM) bites. Uninfected and CHIKV infected *Ae. aegypti* ware allowed to feed upon mouse ears, and total RNA was extracted from biopsies at the indicated times. Real-time RT-PCR was performed to measure expression of the indicated cytokine mRNAs. RNA extracted from ears of mice not exposed to mosquitoes were considered as naïve and assigned an arbitrary value of 1.0, and changes in mosquito-induced cytokine gene expression are expressed as the ratio between mosquito-fed and naïve samples. GAPDH gene was used as a normalizing control. The asterisk denotes a statistically significant difference between the means of naïve and experimental groups (*-*P*≤0·05; **- *P*≤0·001). *N* = 3 per group.

Mosquitoes inject saliva at the bite site during blood feeding to circumvent the host physical barriers and the complex and redundant physiological responses orchestrated by the host's haemostatic and inflammatory systems that have evolved to prevent blood loss and combat infection. Effects of CHIKV infected mosquito saliva on the dermal cell expression of cytokines at the mosquito bite site have not been reported. To characterize the influence of infected mosquito saliva on the first events of CHIKV transmission, cytokine gene expression was measured by real-time RT-PCR in skin biopsies collected at 3 and 6 hours post –feeding (hpf) by uninfected and CHIKV infected mosquitoes (Fig 1) as relative fold difference compared to naïve mice skin biopsies. In the uninfected mosquito bite samples, expression of IL-4 was up-regulated 417 fold and 76.9 fold at 3hpf and 6 hpf, respectively, while the expression of IL-2, IFN-γ and TLR-3 were down-regulated 6.6 fold, 4 fold and 3.7 fold, respectively, at 6 hpf in the same samples. In the CHIKV infected mosquito bite biopsies, expression of IL-4 was up-regulated 567 fold and 604 fold at 3 hpf and 6 hpf, respectively, while the expression ofIL-2, IFN-γ, and TLR-3 were down-regulated 2.1 fold, 1.6 fold and 5.5 fold at 6 hpf, respectively. Importantly, expression of IFN-γ was down-regulated 4.3 fold at 3 hpf in tissues exposed to CHIKV infected mosquito bites (Fig 1). Notably, compared to the uninfected mosquito bites, the expression of IL-4 was 150 fold and 527.1 fold higher in the CHIKV infected mosquito samples at 3 hpf and 6 hpf, respectively (Table 1). Overall, T_H_2 cytokines were significantly up-regulated while T_H_1 cytokines were significantly down-regulated at both study time points.

**Table 1:** Differential expression of cytokines and TLR-3 induced by CHIKV infected mosquito bites.

	Relative fold difference compared to uninfected mosquito bites			
	UIM-3 hpf (naïve)	CIM-3 hpf	UIM-6 hpf (naïve)	CIM-6 hpf
**IL-2**	1	−2.6	1	4.4*
**IL-4**	1	150*	1	527.1**
**IL-10**	1	0.55	1	1.01
**IFN-γ**	1	−2.8*	1	2.3*
**TLR-3**	1	3.64*	1	−1.8

Relative fold differences were calculated by considering uninfected mosquito bites as naïve. (UIM- uninfected mosquito bites; CIM- CHIKV infected mosquito bites).

*- P≤0.05; **- p≤0.001.

### Cytokine responses to needle injected CHIKV

**Figure 2:**
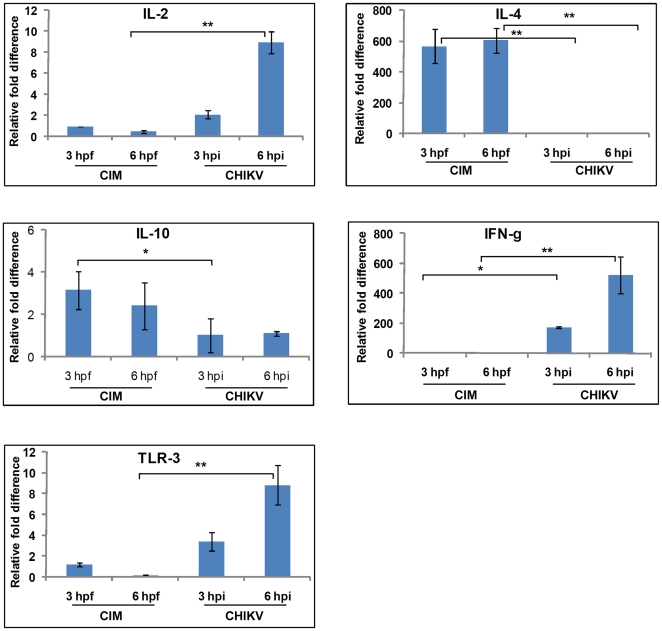
Comparison between CHIKV infected mosquito (CIM) bites and needle injected CHIKV. CHIKV infected *Ae. aegypti* were allowed to feed on mouse ears and total RNA was extracted from biopsies at the indicated times. In parallel, total RNA was extracted from mouse ear biopsies at sites of needle inoculation of CHIKV or medium without virus. Real-time RT-PCR was performed to measure expression of the indicated cytokine mRNAs. RNA extracted from ears of mice not exposed to mosquitoes was considered as naïve for CHIKV infected mosquito bite tissue samples. Medium-inoculated mouse biopsy samples were considered naive for needle inoculated CHIKV samples. Naive samples were assigned an arbitrary value of 1.0, and changes in mosquito-induced cytokine gene expression were expressed as the ratio between mosquito-fed and naïve samples. GAPDH gene was used as a normalizing control. The asterisk denotes a statistically significant difference between the means of naïve and experimental groups (*-*P*≤0·05; **- *P*≤0·001). *N* = 3 per group.

To understand the first immunological events in CHIKV infection without mosquito saliva, we measured the expression of cytokines at sites of intradermal needle-injected CHIKV, using real-time RT-PCR and compared with that of medium injected mouse ear biopsies (naïve). Notably, T_H_1 cytokines were significantly up-regulated while T_H_2 cytokines showed no significant change in their expression (Fig 2). Expression of TLR-3 was up-regulated 3.4 fold and 8.8 fold at 3 hpi and 6 hpi, respectively. Expression of IFN-γ was up-regulated 172 fold and 523.2 fold at 3 hpi and 6 hpi, respectively, and expression of IL-2 was up-regulated 2.1 fold at 3 hpi and 8.9 fold at 6 hpi, compared to naive.

### Cellular recruitment by mosquito saliva

**Figure 3:**
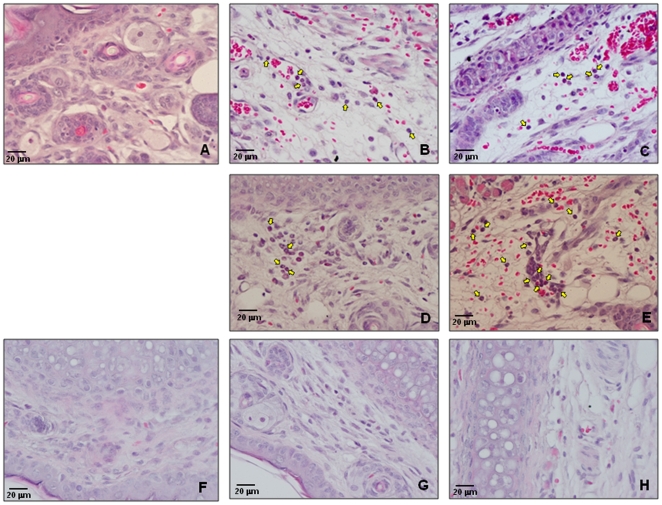
Histopathological changes at the mosquito bite and CHIKV injected sites. Biopsies obtained from mouse ear samples were fixed in 10% neutral formalin and paraffin embedded. Four to five millimeter sections were made and H&E stained. Slides were observed for cellular recruitment at the mosquito bite site or CHIKV injection site. Yellow arrows in the images point to eosinophils. A-uninfected mice (naïve); B- uninfected mosquito bite site (3hpf); C- uninfected mosquito bite site (6 hpf); D-CHIKV infected mosquito bite site (3 hpf); E-CHIKV infected mosquito bite site (6 hpf); F-medium injected site, G-needle injected CHIKV site(3 hpi); H-needle injected CHIKV site (6 hpi). *N = 3* per group.

To further understand the effects of mosquito saliva in arboviral transmission, mosquito bite sites were paraffin embedded, sectioned and H&E stained. Recruitment of eosinophils was observed at both uninfected and CHIKV-infected mosquito bite sites (Fig 3 B, C, D and E). Although, a few neutrophils were observed at the CHIKV-infected mosquito bite sites, eosinophils were present in abundance. Notably, more eosinophil recruitment was observed at CHIKV- infected mosquito bite sites. Histological examination of biopsies taken at the CHIKV-injected site did not show any immune cell recruitment. There were no differences in the cellular population between naïve and CHIKV-injected sites at either 3 or 6 hpi (Fig 3 G and H).

## Discussion

This report provides the first analysis of selected cutaneous cytokine changes in a mouse model of CHIKV infection by mosquito bite. We also describe significant comparative differential cytokine responses of CHIKV transmitted by mosquito bite or CHIKV transmitted by intradermal needle inoculation. Mouse ears exposed to uninfected *Ae. aegypti* bites induced significant levels of IL-4 and suppressed IFN-γ, IL-2, and TLR-3 transcripts. These changes correlated with our earlier findings of similar responses to mosquito bites by BALB/c mice [25]. Interestingly, CHIKV-infected *Ae. aegypti* that fed on mouse ear induced a similar response but with a higher fold induction of IL-4. Significantly, increases of 150 fold at 3 hpf and 527.1 fold at 6 hpf were observed for CHIKV infected bite site biopsies, when compared with uninfected mosquito bite site biopsies (Table 1). Clearly, CHIKV infected mosquito bites prolong suppression of T_H_1 cytokine production, while inducing expression of T_H_2 cytokines. This skewing of host immunity towards a T_H_2 profile at the bite would favor infection and dissemination of CHIKV in the host due to down-regulation of anti-virus T_H_1 cytokines.

Modulation of host immunity towards T_H_2 responsiveness by mosquito saliva is reported to facilitate transmission of both CVV [15] and WNV [16]. It is plausible that CHIKV could have evolved to facilitate upregulation or downregulation of mosquito secretion of salivary proteins/factors that could favor virus replication, transmission and/or persistence in the host.

Previous studies reported that feeding efficiency of *Ae. aegypti* were adversely affected by arboviral [26] or malaria parasite [27] infection. These behavioral and physiologic effects are likely associated with changes in the structure of the salivary glands or in the composition of mosquito saliva. Infection of WNV in the mosquito salivary glands induced distinct morphologic and cytopathologic changes. Salivary gland function and virus transmission efficiency changed during the course of WNV infection due to pathological changes in the mosquito [28], resulting in a differential salivary gland transcript profile [29]. In *Anopheles gambiae*, 57 salivary gland genes were differentially regulated upon *Plasmodium berghei* infection [30].

Understanding the role of mosquito saliva in the earliest stages of CHIKV infection and transmission was highlighted by the dynamics of T_H_1 and T_H_2 cytokine responses when comparing needle inoculated-CHIKV with to CHIKV introduced into the host by mosquito bite. Needle inoculated-CHIKV polarized the host cytokines towards a T_H_1 response with significant up-regulation of IFN-γ and IL-2. Expression of IL-4 and IL-10 did not show significant change in transcript levels following needle inoculation of virus. In contrast, CHIKV infected mosquito bites skewed the host immunity towards a T_H_2 profile with significant IL-4 regulation. These findings clearly demonstrate that CHIKV infected mosquito feeding skews host T-cell immune responses away from a T_H_1 to a T_H_2 phenotype, which in turn can facilitate transmission of CHIKV that might otherwise be inactivated by T_H_1 cytokines.

Toll-like receptors (TLR) have essential roles in the initiation of innate immunity to infectious agents. In mammals, TLR family is composed of at least 12 members and each TLR acts as a primary sensor of conserved microbial components and drives the induction of specific biological responses [31]. Specific recognition of viruses by TLRs has been previously documented. The role of TLR-3 has been implicated in protective immune responses against single stranded RNA viruses such as WNV [32] and double strand RNA viruses such as Lang reovirus [33]. Our data show that needle injected CHIKV significantly up-regulated the transcription of TLR-3. In contrast, the expression of TLR-3 was down-regulated by both uninfected and CHIKV infected mosquito bites.

Expression of IFN-γ is important in defense against RNA viruses by inducing proliferation and differentiation of many cell types, activating the production of cellular proteins that prevent viral mRNA translation; and, enhancing macrophage nitric oxide production [34], [35]. Up-regulation of IFN-γ and TLR-3 in response to needle injected CHIKV suggests their role in protective immune response against this virus. These anti-viral responses are suppressed by CHIKV infected mosquito bites. The prototypical T_H_2 cytokine IL-4 inhibits T_H_1 clonal expansion including the expression of IFN-γ and activation of cytotoxic T-cells [36].

Real-time RT-PCR data reported here correlates with our histological observations. Uninfected and CHIKV-infected mosquito bites recruited eosinophils. This corresponds to up-regulation of IL-4 transcription. Also, CHIKV-infected mosquito bites recruited more eosinophils than uninfected mosquito bite sites at both 3 and 6 hpf. In contrast, needle injected CHIKV samples did not show immune cell recruitment. Also, the cell population looked similar to the naïve sample (Fig 3). It is possible that the resident cells such as keratinocytes, macrophages, and dendritic cells could be responding to virus infection by up-regulating the antiviral IFN-γ and TLR-3 transcription.

In this study, we describe, for the first time, an analysis of selected cutaneous cytokines during CHIKV infection by mosquito bite, compared with that of needle inoculated CHIKV. Our data demonstrate contrasting immune activation in response to CHIKV infection by these two different routes of transmission. This highlights a significant role of *Ae. aegypti* saliva in the earliest events of CHIKV transmission and infection and further confirms the importance of studying a mosquito-borne arbovirus using actual mosquito transmission of the virus, rather than needle inoculation of virus alone. Our study was performed under a controlled environment utilizing mice that were not pre-exposed to CHIKV with the objective of elucidating the earliest immune response to CHIKV infection, and to evaluate the immune response between mosquito- transmitted and needle-injected CHIKV. However, the consequences of CHIKV infection of mice pre-exposed to un-infected/infected mosquito bites or needle-injected CHIKV has not yet been investigated, and it is the subject of our future study.

## Materials and Methods

### Ethics statement

All experiments were conducted in an animal biosafety level 3 (ABSL-3) facility in accordance with a protocol (number: 0912070) approved by the University of Texas Medical Branch (UTMB) Institutional Animal Care and Use Committee (IACUC).

### Animals

Outbred CD-1 strain used in this study mice were obtained from Charles River Laboratories (Wilmington, MA). Mice were cared for in accordance with guidelines of the Committee on Care and Use of Laboratory Animals (Institute of Laboratory Animal Resources National Research Council, Washington, DC).

### Virus

Full length infectious clones of CHIKV that express GFP (CHIKV-LR 5′GFP) [**31**] was used in this study. This infectious clone was produced using the LR2006 OPY1 strain of CHIKV (CHIKVLR), obtained from the World Reference Center for Arboviruses at the University of Texas Medical Branch, Galveston, TX, and readily infects *Ae. aegypti* at a similar rate to the wild type virus, LR2006 OPY1 [37]. The presence of GFP in this clone allowed us to determine CHIKV infection in mosquitoes using epifluorescence microscopy.

### *In vitro* growth of virus

C6/36 *Ae. albopictus*-derived cells were maintained at 28°C in Leibovitz L-15 medium supplemented with 10% FBS, 100 U/mL penicillin, and 100µg/mL streptomycin [31]. Confluent monolayers of C6/36 cells were infected with CHIKV at a multiplicity of infection (moi) of 0.1 by rocking for 1 h at 25°C in 25-cm^2^ flasks. Cells were washed with 5 mL of L-15 medium three times and then 5.5 mL of L-15 medium was added per flask. At day 0 and at 12, 24, 48, 72, and 96 hours post-infection (hpi), a 0.5-mL sample of medium was removed and stored at −80°C. The volume of medium was restored by adding 0.5 mL of fresh medium after each sampling.

### Titrations

Viral samples harvested from cell culture were quantified as tissue culture infectious dose 50 endpoint titers (log_10_ TCID_50_/mL) as described previously [37], [38]. Briefly, 100mL samples of cell culture supernatant medium was pipetted into wells of the first column of a 96-well plate seeded with C6/36 cells, serially diluted in a 10-fold series, and were incubated at 37°C for 7 days with 100 U/mL penicillin, and 100µg/mL streptomycin.

### Mosquito maintenance

The *Ae. aegypti* Higgs White eye strain colony used in this study was maintained within the Biosafety level-3 (BSL-3) insectary at the University of Texas Medical Branch in Galveston. This colony was maintained at 28°C, with relative humidity of 70–75% under a light∶dark cycle of 14hr∶10hr with a 1h crepuscular period to simulate dawn and dusk. Mosquito eggs were maintained on semi-wet filter-paper in a humidified chamber. Eggs were placed into a plastic pan with water of approximately 1-inch depth with a small amount of food (1∶1∶1 powdered laboratory rodent diet, lactalbumin and brewers yeast) added. Under these conditions, larvae developed into the pupae stage in six to seven days. Pupae were removed, sex determined, and transferred into a small cup of water placed in a rearing cage for eclosion. Emerged adults were provided with 10% sucrose *ad libitum* and fed weekly on anaesthetized hamsters as per NIH guidelines for humane use of laboratory animals. Female mosquitoes were starved for 12–24 h prior to blood feeding.

### Mosquito infections

Four to five day old female *Ae. aegypti* were intrathoracically infected with CHIKV-LR 5′GFP (6 log_10_ TCID_50_/mL) using an isolation glove box located in a BSL-3 insectary. All infections were performed in an isolation glove box. Female mosquitoes were cold-anesthetized and intrathoracically injected by using a Drummond 100µl microcapillary needle prepared with a needle puller (Narishige, Tokyo). Approimately 1µl of 6 log_10_ TCID50/mL of CHIKV-LR-5′GFP in L-15 medium containing 10% FBS, 100 U/mL penicillin, and 100µg/mL streptomycin was injected into each mosquito. Mosquitoes injected with only L-15 medium containing FBS and antibiotics were considered as uninfected control mosquitoes. At nine days post- infection, injected mosquitoes were used in the study.

### Real-time RT-PCR to measure differential cytokine gene expression at mosquito bite sites

Twelve individual CHIKV-infected female *Ae. aegypti* was allowed to blood feed on the left ear of 12 two week old CD-1 mice for 30 minutes. The right ears were excluded in our experiments. Fed mosquitoes were then dissected to check for CHIKV infection by observing GFP signal in the salivary glands. Twelve other female *Ae. aegypti* injected with medium alone (uninfected) were allowed to feed on the left ear of 12 additional mice of the same age. In both of these experimental groups, six mice were used for each time point. Three of the mice in each group were used for cytokine expression analysis and the other three were used for histology. Punch biopsies (4 mm) were then obtained from ear bite sites at 3 hpf and 6 hpf, stored in RNALater (Ambion). Trizol reagent (Invitrogen) was used to extract total RNA. Genomic DNA contamination was eliminated by DNAse treatment. Total RNA was measured using a NanoDrop 1000 (Thermo Scientific) and RNA quality was analyzed by denaturing gel electrophoresis. First strand cDNA was synthesized from 1 µg total RNA using a Retroscript 1^st^ Strand cDNA synthesis kit (Ambion) and subsequently used as template for real-time RT-PCR analysis. Real-time RT-PCR amplifications were performed using RT^2^Real-Time™ SYBR Green/Fluorescein PCR master mix (SABiosciences) in an iCycler (BioRad). The primers used in this experiment are listed in Table 2. Typically, PCR was performed by heating to 95°C for 10 min to heat-activate the HotStart Taq DNA Polymerase followed by 40 cycles of 15 sec at 94°C then 60 sec at 60°C. All reactions were performed in triplicate. Each time point sample had 3 biological replicates. GAPDH mRNA was used as a normalizing standard and RNA from mosquito non-exposed ear biopsies were considered as naïve and assigned an arbitrary value of 1.0. Changes in mosquito bite-induced cytokine gene expression were calculated as the ratio between mosquito bite and naïve samples.

**Table 2:** Primers used for real-time RT-PCR.

	NCBI sequence ID	Primers (5′ to 3′)
**GAPDH**	NM_001001303	TTGAGGTCAATGAAGGGGTC TCGTCCCGTAGACAAAATGG
**IL-2**	NM_008366	GTCAAATCCAGAACATGCCG AACCTGAAACTCCCCAGGAT
**IL-4**	NM_021283	CGAGCTCACTCTCTGTGGTG TGAACGAGGTCACAGGAGAA
**IL-10**	NM_010548	TGGCCTTGTAGACACCTTGG AGCTGAAGACCCTCAGGATG
**IFN-γ**	NM_008337	GAGCTCATTGAATGCTTGGC GCGTCATTGAATCACACCTG
**TLR-3**	NM_126166	ATAGGGACAAAAGTCCCCCA ATGATACAGGGATTGCACCC

### Cytokine response to needle injected CHIKV

Ten micro litres of CHIKV containing 3 log_10_ TCID_50_ were intradermally injected into the left ear of CD-1 mice. The same volume of medium was injected intradermally in to mice serving as a naïve control. Punch biopsies (4 mm) were obtained from the injection sites at 3 hpi and 6 hpi. Total RNA, first strand cDNA synthesis and cytokine real-time PCR were performed as described above. All reactions were performed in triplicate. Each time point sample had 3 biological replicates. GAPDH mRNA was used as a normalizing standard and RNA from the medium injected ear biopsies was considered as naïve and assigned an arbitrary value of 1.0. Changes in mosquito bite-induced cytokine gene expression were calculated as the ratio between mosquito bite and naïve samples.

### Statistics

Statistical analyses were preformed with Graph Pad 4.0 Prism software. One way nonparametric ANOVA followed by Tukey post test was performed [39].

### Histology

Tissues were processed for histology as described by Ziegler et al. (2008). Briefly, 4 mm ear biopsies obtained from each mouse were fixed in 10% neutral-buffered formalin for 36 hours; transferred to 70% ethanol prior to embedding, sectioning at 4 to 5 µm and staining with hematoxylin and eosin (H&E).
